# Cretaceous Small Scavengers: Feeding Traces in Tetrapod Bones from Patagonia, Argentina

**DOI:** 10.1371/journal.pone.0029841

**Published:** 2012-01-09

**Authors:** Silvina de Valais, Sebastián Apesteguía, Alberto C. Garrido

**Affiliations:** 1 CONICET - Instituto de Investigación en Paleobiología y Geología, Universidad Nacional de Río Negro, Fisque Menuco (General Roca), Río Negro, Argentina; 2 CONICET - Fundación de Historia Natural “Félix de Azara”, CEBBAD, Universidad Maimónides, Buenos Aires, Argentina; 3 Museo Provincial de Ciencias Naturales “Prof. Dr. Juan A. Olsacher” – Dirección Provincial de Minería, Zapala, Neuquén, Argentina; University of Maryland, United States of America

## Abstract

Ecological relationships among fossil vertebrate groups are interpreted based on evidence of modification features and paleopathologies on fossil bones. Here we describe an ichnological assemblage composed of trace fossils on reptile bones, mainly sphenodontids, crocodyliforms and maniraptoran theropods. They all come from La Buitrera, an early Late Cretaceous locality in the Candeleros Formation of northwestern Patagonia, Argentina. This locality is significant because of the abundance of small to medium-sized vertebrates. The abundant ichnological record includes traces on bones, most of them attributable to tetrapods. These latter traces include tooth marks that provde evidence of feeding activities made during the sub-aerial exposure of tetrapod carcasses. Other traces are attributable to arthropods or roots. The totality of evidence provides an uncommon insight into paleoecological aspects of a Late Cretaceous southern ecosystem.

## Introduction

The available information about fossil tetrapods is biased towards their hard parts (i.e., bones and osteoderms) and the interaction of these organisms with the substrate (i.e., tracks). Evidence of biotic interactions between tetrapods and other organisms, particularly other tetrapods, are rarely reported and deciphering such evidence represents a challenge to understanding the fossil record. Evidence such as animal-animal interactions are best interpreted through examination of the ichnological record, which has the potential to elucidate functional aspects of the ecosystem. Actually, bone modification features are capable of revealing the injuries caused by agonistic behavior or predator attack, and also scavenging activities on skeletal remains (e.g., [1]), where bioerosive activity becomes relevant [2]. Surprisingly, although these features are commonly found in the fossil record, they are clearly underrepresented in the scientific literature and, when mentioned, are usually not detailed or figured.

During the last few years, a renewed interest in these phenomena has begun to change this situation (e.g., [3], [4], [5], [6]). However, due to differences in the fossil representation or in searching efforts, the vast majority of these reports are often focused on the northern hemisphere (e.g., [1], [7], [8], [9], [10], [11]), whereas few concern southern hemisphere ecosystems (e.g., [12]).

**Figure 1:**
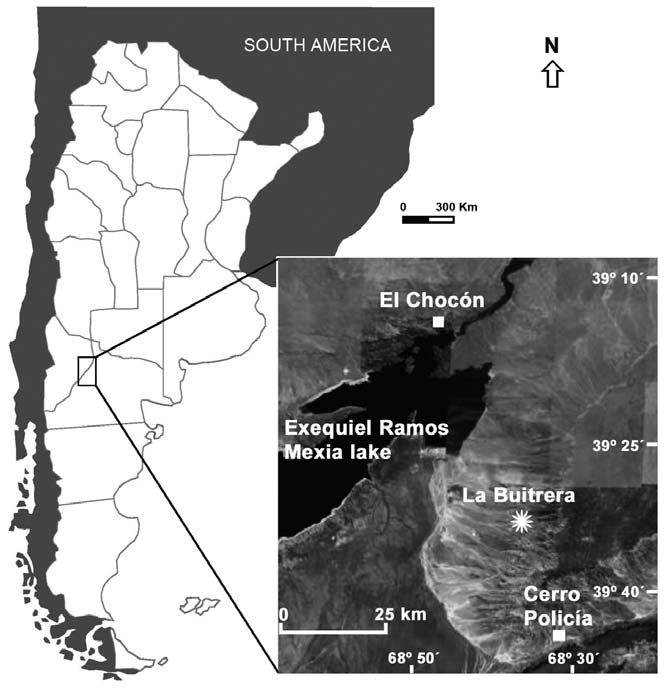
Map of northwestern Patagonia showing the new locality (La Buitrera). El Chocón and Cerro Policía towns are also shown. Red deposits of the Upper Cretaceous are widely distributed eastward from the Andean Range of Argentina. (One-column width, already sized figure).

The Upper Cretaceous strata from northern Patagonia, Argentina, have demonstrated an unusual potential for the preservation of large dinosaurs (e.g., [13], [14], [15]), but also of small and medium-sized tetrapods (e.g., [16], [17], [18]). The locality known as La Buitrera, located 32 km northwest of Cerro Policía (Río Negro Province, Patagonia, Argentina) (Fig. 1), has yielded numerous taxa of mainly “medium-sized” tetrapods that preserve superb histological details both in their bone structure and on their surfaces. The early Late Cretaceous sandstones of the Candeleros Formation [19] that outcrop at La Buitrera locality have an excellent preservation potential due to their deposition under brief periods of subaerial exposure, as evidenced by several levels of paleosols as well as abundant bioerosive structures on the surfaces of tetrapod bones.

The aim of this contribution is to describe these trace fossils and, when possible, to analyze and interpret the possible tracemakers and consider some insights on the ecological relationships of different components of this Cretaceous ecosystem.

### Geological Setting

The bones bearing the trace fossils were collected in sandstones from the upper levels of the Candeleros Formation (Cenomanian), the basal unit of the Rio Limay Subgroup, Neuquen Group [20], [21]. In the south area of the Neuquén Basin, this lithostratigraphic unit is overlain by the Huincul Formation, and unconformably underlain by the Lohan Cura Formation [22], [23].

The La Buitrera fossiliferous locality is located 32 km NW of Cerro Policía, northern Río Negro Province, Argentina. The main fossiliferous area is about 4 km^2^ and contains at least four trace-bearing levels, surrounded by 40 m-tall cliffs in an arid landscape not far from the Ezequiel Ramos Mexía dam, along the boundary between the Neuquén and Río Negro provinces, Argentina (Fig. 1; see Figure S1).

Important fossils were recorded northwards from the lake, close to the town of El Chocón (e.g., [24], [25], [26]). Southward, on the opposite shore, the La Buitrera fauna shows a preservational bias toward small to medium-sized forms, thereby becoming highly informative within the framework of Mesozoic tetrapods. Furthermore, as an interesting taphonomic aspect, it is noteworthy that most specimens of these small tetrapods are articulated or slightly disarticulated [27]. In several cases, recent subaereal exposure resulted in new disarticulation of the bones, spreading them over the ground before discovery. This is evidenced by several specimens in a slight degree of disarticulation (i.e., MPCA 380, a single snake specimen associated but spread over two square meters).

Sedimentological evidence at La Buitrera exhibits distinct lithofacial features when compared to coeval localities (i.e., El Chocón, Neuquén Province), probably as a response to different environmental conditions of sedimentation [27]. The Candeleros Formation at La Buitrera locality conform a succession 95 m thick, composed by quartzitic, coarse to medium-grained sandstones, sabulitic and conglomerate lenses and frequent intercalation of siltstones and mudstones. The dominant colour of their outcrops varying between purple, dark red and brownish. This succession is characterized by the presence of channelized bodies with lateral accretion macroforms, suggesting the development of a meandering fluvial system. Recent studies [28] indicated that these ancient rivers ended in a closed playa-lake system, linked to the Picún Leufú Sub-basin. The overbank sediments are well developed, mainly represented by levee, crevasse channels, and crevasse splay deposits. Paleosols (Figure S1) are poorly developed and are typified by the presence of pedogenic caliche and scarse rizholits. Discontinuities in the sedimentation rate represent different events of periodical flooding followed by a short aerial exposure, as evidenced by the limited attack on the carcasses [27].

On the other hand, at the El Chocón area, the Candeleros Formation exceed 150 m thick. This succession is composed by quartzitic sandstones, wackes, and mudstones, with thin intercalations of evaporites (gypsum) and tufites. The studies realized by Garrido [28] allow understanding it as a depositional environment dominated by facies of terminal fans, associated to playa-lakes, aeolian dunes and interdune deposits. The paleosols are poorly developed and are represented by both calcareous grounds developed on the dune deposits and vertisols over the muddy deposits of sheet floods.

The dissimilar paleoenvironmetal particularities that occurred at the El Chocón and La Buitrera areas could have contributed to differences in the distribution and habitat preferences of the biota, as well as in the taphonomic modes of preservation. Two main taphonomic modes have been observed in the La Buitrera locality: 1) red fine-grained sandstones containing well articulated specimens, representing immature paleosols developed in water-satured levee deposits, and 2) yellow medium-grained sandstones with disarticulated specimens, representing lateral accretion deposits and the subaerial top of point bars [27], [29].

The ichnological record of this locality is quite rich. It includes the feeding traces described here, along with coprolites and abundant tubular structures, most probably paleo-burrows, which can be appreciated as they pierce the walls of the cliffs. Both the coprolites and the tubular structures will be described elsewhere.

## Materials and Methods

The trace fossils examined are preserved as negative epichnia (convex) on the surface of tetrapod bones, mainly sphenodontids, crocodyliforms and theropod dinosaurs. Most of the traces are present in isolated and/or disarticulated specimens, but also in articulated skeletons. Although common on long bones, they are also present on vertebrae and cranial bones. As this material did not require technical preparation, the risk of being artifacts of preparation is very low. Specimens are housed in the vertebrate collection of the Museo Provincial “Carlos Ameghino”, Cipolletti, Río Negro Province, Argentina, under the acronym MPCA. Each of the fossil bones and their corresponding traces are compiled in the Table 1.

**Table 1:** Summary of the trace fossils on bones.

Group	Specimen	Trace-bearing bone
1	MPCA 470-1	Fragment of araripesuchid fibula or radius
	MPCA 470-2	Fragment of sphenodontid skull
	MPCA 470-3	Fragment of neural arch, indet
	MPCA 470-4	Fragment of sphenodontid jaw
2A	MPCA 470-6	Araripesuchid ischia (in the same bone than MPCA 470-11)
	MPCA 470-7	Sphenodontid maxilla
	MPCA 470-8	Sphenodontid dorsal centrum vertebrae
	MPCA 470-9	Fragment of sphenodontid skull
	MPCA 470-10	Araripesuchid tibia or ulna
	MPCA 470-11	Araripesuchid ischia
	MPCA 470-12	Araripesuchid or sphenodontid coracoid
	MPCA 470-13a-b	Right dentary of *Buitreraptor gonzalezorum* Makovicky et al. 2005, MPCA 245
2B	MPCA 470-5	Fragment of rib, indet

Trace fossils on bones are abundant among the fossils from La Buitrera. However, in order to avoid different preservational varieties, we will pay special attention only to those specimens with well-preserved morphological features. In this context, we have left out those traces with overprint of different generations of related traces, weathering features, or those too shallow to be evaluated. On the other hand, although the morphological types of the trace fossils are distinctive enough from all published ichnotaxa, the available material is too scarce to warrant the proposal of new ichnotaxa.

The trace fossils were photographed with a digital camera both at normal view (under low-angle illumination) and under a SEM JSM-6460LV with no gold cover. They were observed using an optical microscope Zeiss Stemi SV11 and a trinocular loupe ARCANO ZTX-T.

To analyze the association between the ichnofossils and their possible tracemakers, the whole fossil record from La Buitrera and surrounding localities was studied. From the known fauna, attention was focused on skeletal regions capable of producing marks on bones, such as teeth, jaws and claws, but also on the morphology and spatial distribution of the traces. Paleobiological aspects of the taxa were considered in the analysis.

## Results

### Description of the traces

On the basis of their morphology and spatial arrangement, all the trace fossils can be separated into two main groups, informally named as Networks and Furrows, The latter is in turn subdivided into Parallel Furrows and Pits (Table 1).

#### Networks

**Figure 2:**
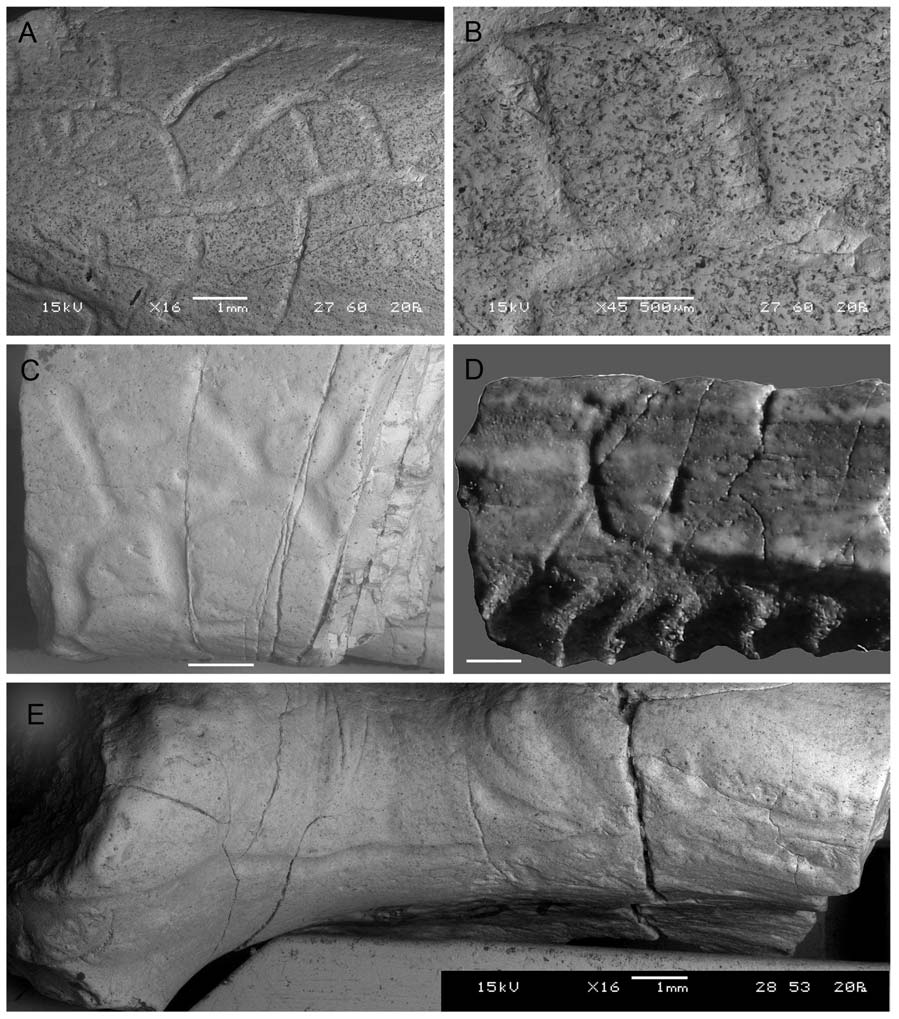
Trace fossils included in the Group 1. A. MPCA 470-1; B. detail of MPCA 470-1; C. MPCA 470-2; D. MPCA 470-4; E. MPCA 470-3. Scale bars represent 1 mm in A, C–E, and 0,5 mm in B. (Two-column width, already sized figure).

The networks are composed of interconnected grooves with different degree of development, represented by specimens MPCA 470-1 to -4 (Fig. 2).

The best-preserved specimen is MPCA 470-1 (Figs. 2A, B). It comprises a central groove with several secondary grooves of variable length that branch off at different angles from the main axis (45° to 80°) and follow straight to curved paths. The grooves display a rather constant width of 0.19 mm in average, and ranging from 0.16 mm to 0.25 mm. The branching points are commonly represented by a minute hiatus in the bone bioerosion. The grooves are shallow, scattered, and U-shaped in cross-section, with a smooth surface. Some channels display marks distributed perpendicular to the main axis of the groove, but they are probably not related to the trace origin (Fig. 2B).

Other specimens of this group are represented by less developed networks, commonly with fewer branches than MPCA 470-1. Considering the shallow relief of the structures and the larger width of the grooves in specimens MPCA 470-2 (Fig. 2C) and MPCA 470-4 (Fig. 2D), these are probably weathered specimens. Both samples have barely visible grooves up to 0.5 mm wide. MPCA 470-3 is the least-developed trace, composed of a relative long groove with three short branches, only visible under special conditions of illumination (Fig. 2E).

#### Furrows

**Figure 3:**
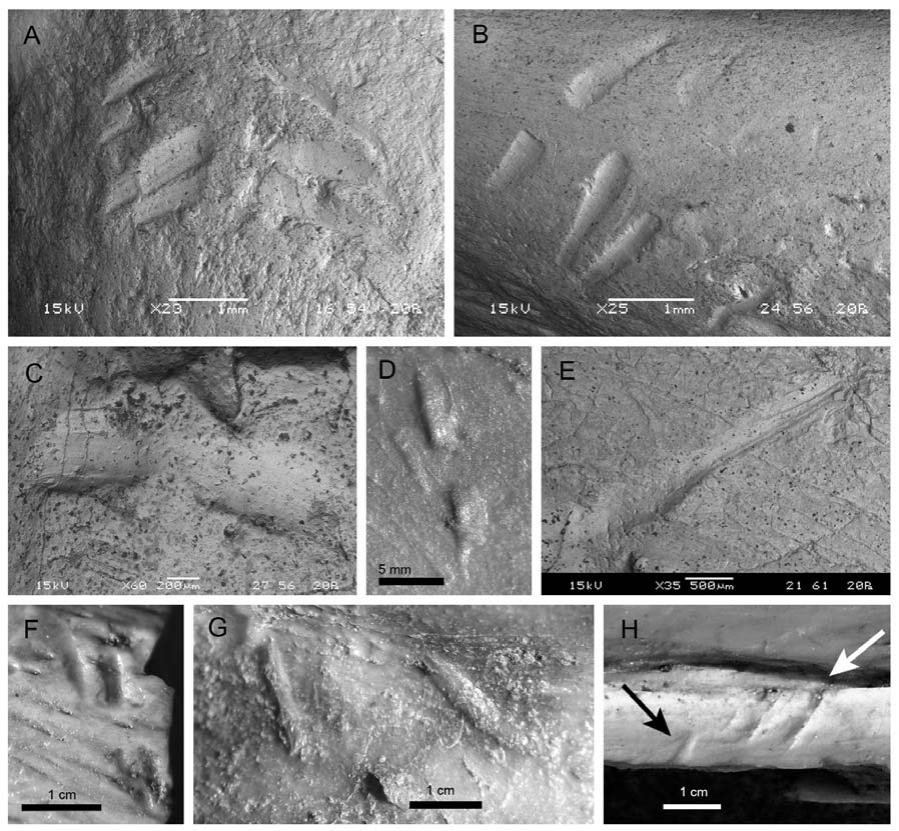
Trace fossils included in the Subgroup 2A. A. MPCA 470-11; B. MPCA 470-12; C MPCA 470-10; D. MPCA 470-6; E. MPCA 470-7; F. MPCA 470-9, G. MPCA 470-8, H. MPCA 470-13a (white arrow) and MPCA 470-13b (black arrow) preserved in *Buitreraptor gonzalezorum* Makovicky et al. 2005. Scale bars represent 1 mm in A, B, E–G, 0,5 mm in D, and 0,2 mm in C. (Two-column width, already sized figure).

These traces represent isolated parallel to subparallel furrows, often arranged in pairs (Fig. 3). Based on the spatial arrangement of the furrows, the specimens can be separated into two informal subgroups: Parallel Furrows and Pits.

The Parallel Furrows include trace fossils composed of one or more, wide, straight furrows, represented by specimens MPCA 470-6 to -13a-b (Fig. 3). The furrows can be isolated (Figs. 3D,G), arranged in parallel rows (e.g., Fig. 3F), in opposite pairs (Fig. 3C,D), or as a combination of the two latter types (Figs. 3A,B,E). Each mark ranges from 0.3 to 0.5 mm in width, and 0.7 mm to 2.8 mm in length. When arranged in parallel rows, the distance between the successive furrows is up to 1.6 mm. The more closely packed cases are in slight contact. When arranged in opposite pairs, both traces of the pair display a similar length and, although approaching each other, they never meet. The surface of the traces is smooth or lightly striated parallel to the main axis of each mark.

Specimens MPCA 470-11 and MPCA 470-12 possess at least six furrows, arranged in parallel and opposite pairs (Figs. 3A–B). MPCA 470-11 is the more complex specimen, as it shows three parallel and opposite pairs, with the first furrow of the left row composed of a double lateral mark and the second furrow of a double aligned mark. The furrows on the right row display an elongation in the external ends (Fig. 3A). MPCA 470-12 shows three parallel furrows and three opposite pairs, although the right furrow of the third pair is so smoothly impressed that it is only visible under special light conditions (Fig. 3B). MPCA 470-9 and MPCA 470-13a have a distribution similar to the preceding specimens but with two parallel and opposite pairs (Fig. 3F–H). MPCA 470-13a, together with MPCA 470-13b (see below), are the only trace fossils preserved on a bone of an articulated specimen, *Buitreraptor gonzalezorum* Makovicky, Apesteguía and Agnolín 2005 (MPCA 245). They are in the lateral surface of the right dentary, near the base of the teeth (Fig. 3H) Specimens MPCA 470-6 (Fig. 3D) and MPCA 470-10 (Fig. 3C) display furrows in opposite pairs, whereas MPCA 470-8 (Fig. 3G) displays the furrows arranged obliquely with respect to each other, probably as consequence of being preserved on a curved surface.

The other two specimens included in this subgroup are numbered MPCA 470-7 and MPCA 470-13b, and each is composed of a single isolated furrow (Figs. 3E,H). MPCA 470-7 is a 2.8 mm-long striated channel, with more deeply impressed striae close to one of the ends, which displays a radial crushed surface (Fig. 3E). MPCA 470-13b is 0.7 mm long, with the trace beginning right at the base of a tooth of *Buitreraptor gonzalezorum* Makovicky, Apesteguía and Agnolín 2005.

**Figure 4:**
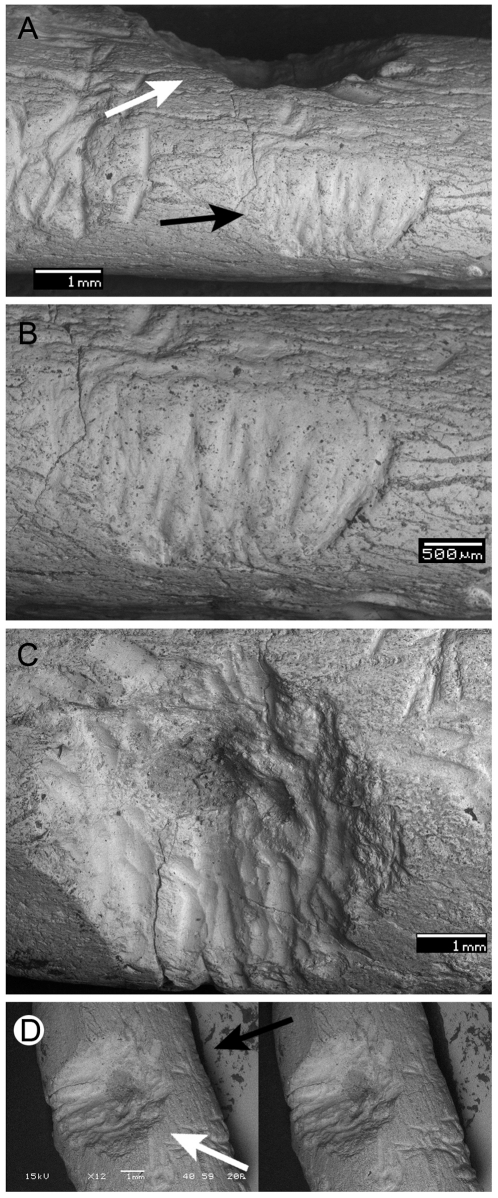
Trace fossil included in the Subgroup 2B, MPCA 470-5. A. general view of the trace fossil; B. detail of the secondary associated trace to the main pit; C. detail of the main subconical pit; D. stereogram of the main subconical pit. The white arrows indicate the main pit, the black arrows indicate the secondary trace. Scale bars represent 1 mm in A, C and D, and 0,5 mm in B. (One-column width, already sized figure).

The Pits are represented only by specimen MPCA 470-5, and composed of two associated traces (Fig. 4). The major trace includes superposed furrows radiating from a central point, resulting in a subconical pit. It is located on the diaphysis of a long bone, probably a humerus. Although the high overprinting of the furrows makes individual identification difficult, it is possible to interpret that they display the same general morphology as those included in parallel furrows. The subconical pit is a nearly circular mark of 5 mm diameter and about 1 mm depth in the center (Figs. 4A,C,D). The secondary trace associated with the pit is located at the same height, at nearly 90° with respect to the radial pit, following the curve of the bone (Figs. 4A,B). The set of marks is about 3 mm long and 1.8 mm wide. It is composed of the parallel disposition of narrower furrows, each about 0.1 mm wide, perpendicular to the major axis of the bone.

### Vertebrate Fauna from La Buitrera locality

The diverse vertebrates fauna of La Buitrera includes rather complete, articulated, three-dimensionally preserved skeletons of saurischian dinosaurs [18], [30], crocodyliforms [31], chelid turtles, basal snakes [32], lizards [33], sphenodontid lepidosaurs [17], dryolestoid mammals, and ceratodontiform dipnoans [34] (Figure S1).

Among the components of this fauna, the most relevant for this contribution are the most abundant taxa, i.e., the sphenodontid lepidosaurs, mainly represented by herbivorous eilenodontines [17]. However, there are also remains of a small sphenodontid, perhaps with insectivorous characteristics [35]. Although sauropod teeth, turtle beaks, lizard teeth, snake teeth and dipnoan tooth-plates are capable of marking bones, they were left out of the search for candidates for marking bones because of their size, shape, or presumed behavioral use. Sauropods are herbivorous and exceedingly large, chelid turtles are piscivorous and devoid of teeth, carnivorous lizards have weak jaws to mark bones, and dipnoans eat snails and do not bear teeth but dental plates. So, the main aspirants for being tracemarkers of the tooth marks described here are presumably crocodyliforms, dromaeosaurids, snakes and mammals.

## Discussion

The subaerial exposure of the bones from La Buitrera locality [27] is shown by their recurrent attack by terrestrial tetrapods. Although the slight disarticulation of the skeletons, the lack of some specific parts (i.e., chevrons and ribs in snakes, or most of the spenodontian tails) could have occurred either under water or on the land surface, the crushing and wear of in some sphenodontid skulls as well as the uncrushed preservation of delicate tridimensional skeletons suggest terrestrial deposition [27]. The presence of ferruginous spots in the rock as a main trait in the edaphized levels and the low degree of horizon development suggest immature paleosols exposed to frequent, perhaps seasonal, flooding episodes. The recurrent paleosols coincide with the fossil-bearing layers, and suggest periods of temporary environmental stability [23], [27].

The frequent recovery of material in death position provides some clues about the way these animals died. Articulated skeletons are positioned in an oblique to vertical position with respect to the stratum level, with the tail inside the rock and a strongly worn allostotic skull roof or, in other cases, with the skull sectioned (e.g., MPCA 306, 315). This resembles the escape thanatocoenosis described for dinosaurs in Cretaceous strata of the Gobi Desert, Mongolia [36]. Thus, it is possible that many of the specimens from La Buitrera could actually represent part of an escaping thanatocoenosis. Considering that the materials are distributed in at least four levels, it is possible that it represents repetitive flooding events [27].

Additionally, the temporary preburial exposure of the bones is supported by the putative terrestrial nature of the proposed trace-makers. However, considering that most skulls retain articulated with the lower jaws, the exposure time was not excessive. Actually, most of the disarticulation results from recent weathering processes that spread the previously articulated material in the loose sand [27].

Most of the trace fossil-bearing bones correspond to isolated elements. Conversely, partially to fully articulated skeletons are almost devoid of feeding traces, which seems to indicate a faster burial. This might have kept them safe from micro- and macro-scavenging. A trace fossil present on the lower jaw of *Buitreraptor gonzalezorum* Makovicky et al. 2005; MPCA 470-13, Fig. 3H), included in the Parallel Furrows, is an exception.

The bone remains mentioned here show no evidence of digestion, regurgitation or excretion (e.g., preservation in pellets or coprolites). This condition suggests that the tracemarkers did not ingest a large part of the meat and bones, but only scratched the surface, and therefore were of small size. This excludes the large species of crocodyliforms and saurischian dinosaurs. Additionally, the former are also disqualified due to their blunt teeth.

The specimens included in the Networks group are composed of interconnected grooves. Like many trace fossils, their origin is not easy to assess. Although not fully understood, they are morphologically similar to root traces (i.e., tape-like structures). However, root traces caused by acid attack or other processes on the hard substrate result in light, occasionally branching traces. They show a non-prominent negative relief [37] or dendrite traces, with main and bifurcated branches [38]. Despite the extension of paths is short to evaluate width changes they display a quite stable width and no channel of different degrees. Further, on the basis of the regular width, they seem to be related to invertebrate feeding structures. The animal would have moved as it ate the periostium of the surface of the bones, thus forming these net-like structures. Although there is no evidence of mandibular scratch marks on the grooves, other traces related to insect activity lack them too. An example is the distinct trace fossils characterized by shallow, radiating, and often paired grooves that have been frequently mentioned in the literature as “star-like pit marks” (e.g., [4], [5], [39], [40], [41], [42]). They are related to the activity of insects, mainly dermestids [42], [5] and termites [41], [43].

These structures are radial in shape, like the trace fossil representing the pits, but differ from the main cavity of MPCA 470-5 in having fine, usually paired striae that form a shallow depression. Unlike the star-shaped pit marks, the furrows composing the parallel furrows and the pit are wider with regard to the former, and not paired.

The specimens forming the parallel furrows, composed of two opposite and parallel marks, are similar to a trace fossil considered as a bite mark (TMP 2007.036.0002) [6]. However, the specimen from the Late Cretaceous of Alberta differs from the marks included herein by having one parallel pair substantially longer than the opposite pair.

Among the fauna of the La Buitrera,small species of crocodyliforms, dromaeosaurids and mammals bear teeth able to mark furrows in bones.

### Crocodyliforms

This is one of the most abundant groups. The study of the La Buitrera fauna has shown the existence of at least five types of crocodyliforms, four of them are omnivorous forms related to *Araripesuchus* [31] and the fifth a large (unpublished) carnivorous neosuchian. The latter has conical teeth, rounded in cross-section, more capable of piercing and biting than clean cutting. A juvenile specimen of the neosuchian is a possible candidate for making the wide furrows included in Group 2, and even pits on the bone surfaces.

### Dromaeosaurids

This group commonly shows very sharp and mediolaterally compressed teeth. Laurasian dromaeosaurids show well-developed denticulated carinae along both margins of the tooth, with a uniform denticles size (e.g., *Dromaeosaurus* Currie et al. 1990), or restricted in some large teeth to only the posterior border (e.g., *Deinonychus*, *Velociraptor* and *Saurornitholestes*; [44], [45], [46]). The teeth of the unenlagiine dromaeosaurid known from La Buitrera, *Buitreraptor gonzalezorum*, are very small, mediolaterally compressed, lightly striated, and completely devoid of denticles [18]. The expected biting mark would consist of parallel furrows with a gap of 1 to 2 mm between them, corresponding to different crowns. This distribution matches the parallel furrows.

### Snakes

This group of lepidosaurs, with an extensive Cretaceous history in southern continents, is abundant in La Buitrera [31]. Snakes bear small and very sharp teeth that could easily produce parallel scratches in the bone surface when capturing or swallowing the prey, but not scavenging. These marks would agree with the parallel furrows.

### Mammals

There are at least two different kinds of mammals at La Buitrera, here informally named taxa 1 and 2. Taxon 1 corresponds to a dryolestoid with very large caniniforms and procumbent incisiforms. Taxon 2 is a form with rather homodont cylindrical teeth devoid of enamel and procumbent incisiviforms. Since both have large and procumbent incisiforms, is not possible to ascertain from the analysis of tooth marks which of them could be responsible for the traces. A detailed study of the traces and dentitions will be necessary to confirm the precise identity of this trackmaker. However, mammals are the only group with long and small incisiforms capable of strongly gnawing the bones and leaving the opposed marks with squared ends and the pit formed by the intense gnawing of this kind of teeth.

Some records of trace fossils made by mammals have been previously mentioned (e.g., [47], [6], [48]). However, they do not display the morphology of the marks from La Buitrera and certainly correspond to different mammalian lineages.

The known scavenger activity carried on by mammals, and the tooth distribution and morphology in agreement with the abundant trace fossils suggest that an important part of the micro-scavenger activity in the studied locality was developed by Cretaceous mammals.

### Conclusions

Since its discovery, the La Buitrera locality has added an important amount of knowledge to southern Cretaceous terrestrial ecosystems, especially due to its bias toward small to medium-sized forms. The preserved ichnological assemblage provides an uncommon view of quite peculiar feeding trace fossils on the bones of several reptiles. At least three kind of trace fossils are recognized here. The first group are the networks, which are referred to an unknown producer, probably an arthropod or root traces. The other two groups display different spatial distributions of furrows, related to tetrapod activity, including crocodyliforms, snakes, mammals and dromaeosaurid theropods. In particular, specimens MPCA 470-5 to 13 are rather informative since they are coherent with vertebrate taxa currently recognized in La Buitrera. The carcass profit is today developed by several groups of animals, mainly insects and mammals. The finding that mainly mammals were scavenging on the carcasses in the La Buitrera fauna extends the evidence for this adaptative zone to Mesozoic times. The same activity is today played by several mammalian lineages, especially rodents, insectivores and the cingulate edentates [49], which do the same today by rasping carcasses to obtain cartilage and periosteum (from [50]). This seems to be an optimal activity for the procumbent incisiforms of dryolestoids, showing the long stability of scavenger behavior in mammals as an optional or regular food resource since at least early Late Cretaceous times. However, it must be noted that non-mammalian marks also found on the skeletons include possibly crocodyliforms, dromaeosaurids and snakes, the latter marking the bones during hunting and swallowing, not scavenging.

Feeding traces are able to show the interaction between individuals, commonly from different taxa in the fossil record. As long as new information on bone bioerosion arises, along with a better classification of these kinds of relatively common traces in the fossil record, new insights will be revealed for understanding the paleobiology and the paleoecological interactions of the tracemakers, as well as taphonomic constraints.

## Supporting Information

Figure S1 Stratigraphic column at La Buitrera locality showing the procedence levels of specimens distributed along the Candeleros and Huincul formations.
(JPG)
