# Supplementary figures and images for: Cretaceous Small Scavengers: Feeding Traces in Tetrapod Bones from Patagonia, Argentina
Source: PLoS One. 2012 Jan 9;7(1):e29841. doi: 10.1371/journal.pone.0029841 (PMC3253797; doi:10.1371/journal.pone.0029841)

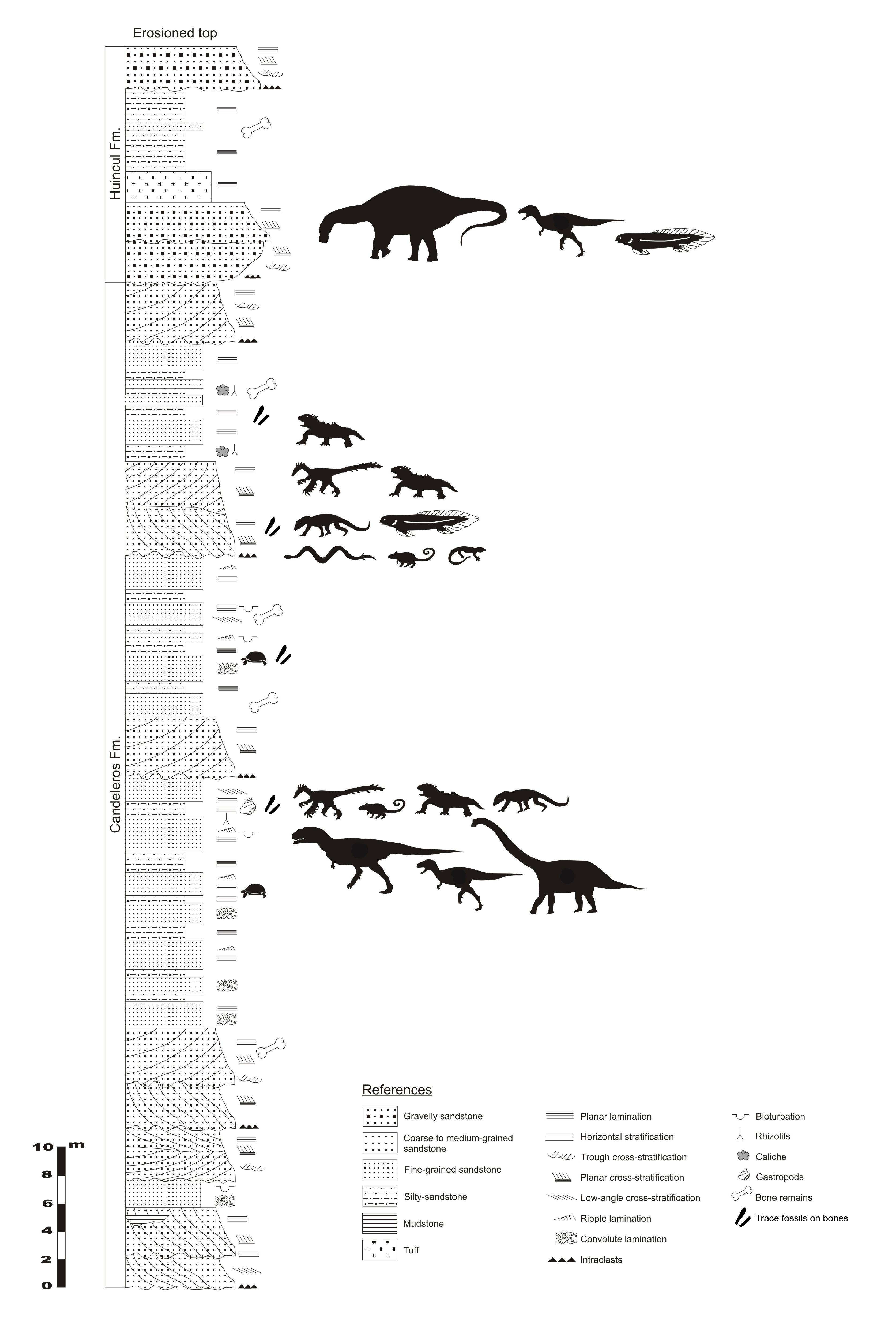

Supplement: Figure S1 — Stratigraphic column at La Buitrera locality showing the procedence levels of specimens distributed along the Candeleros and Huincul formations. (JPG) [file pone.0029841.s001.jpg]
